# Effects of medwakh smoking on salivary metabolomics and its association with altered oral redox homeostasis among youth

**DOI:** 10.1038/s41598-023-27958-w

**Published:** 2023-02-01

**Authors:** K. G. Aghila Rani, Nelson C. Soares, Betul Rahman, Hamza M. Al-Hroub, Mohammad H. Semreen, Sausan Al Kawas

**Affiliations:** 1 Sharjah Institute for Medical Research, University of Sharjah, P.O. Box 27272, Sharjah, United Arab Emirates; 2 Department of Medicinal Chemistry, College of Pharmacy, University of Sharjah, P.O. Box 27272, Sharjah, United Arab Emirates; 3 Department of Human Genetics, National Institute of Health Doutor Ricardo Jorge (INSA), Lisbon, Portugal; 4 Department of Preventive and Restorative Dentistry, College of Dental Medicine, University of Sharjah, Sharjah, United Arab Emirates; 5 Department of Oral and Craniofacial Health Sciences, College of Dental Medicine, University of Sharjah, Sharjah, United Arab Emirates

**Keywords:** Biological techniques, Risk factors, Chemistry

## Abstract

The use of alternative tobacco products, particularly medwakh, has expanded among youth in the Middle East and around the world. The present study is conducted to investigate the biochemical and pathophysiological changes caused by medwakh smoking, and to examine the salivary metabolomics profile of medwakh smokers. Saliva samples were collected from 30 non-smokers and 30 medwakh smokers and subjected to metabolomic analysis by UHPLC-ESI-QTOF-MS. The CRP and Glutathione Peroxidase 1 activity levels in the study samples were quantified by ELISA and the total antioxidant capacity (TAC) by TAC assay kits. Statistical measurements and thorough validation of data obtained from untargeted metabolomics identified 37 uniquely and differentially abundant metabolites in saliva of medwakh smokers. The levels of phthalate, L-sorbose, cytosine, uridine, alpha-hydroxy hippurate, and L-nicotine were noticeably high in medwakh smokers. Likewise, 20 metabolic pathways were differentially altered in medwakh smokers. This study identified a distinctive saliva metabolomics profile in medwakh smokers associated with altered redox homeostasis, metabolic pathways, antioxidant system, and CRP levels. The impact of the altered metabolites in medwakh smokers and their diagnostic utility require further research in large cohorts.

## Introduction

The use of alternative tobacco products (ALTP) is rampant among the youth in the Middle East and currently spreading worldwide. The last two decades have witnessed a tremendous rise in the use of ALTP including dokha (medwakh), shisha (hooka), e-cigarettes, smokeless tobacco, chewing tobacco, and snuff among young adults between 18 and 25 years of age^1,2^. Tobacco smoking disturbs the oral microbiome via immunosuppression, oxygen deficiency, antibiotic effects, and other likely mechanisms^3^. The subgingival and supra gingival microbiota composition in smokers of various tobacco products including medwakh, shisha, and cigarette was reported in our previous studies^4,5^. It has been reported that the microbiome of these types of tobacco smokers was altered in both supra and subgingiva, making them more susceptible to severe periodontal disorders^4^ and dental caries^6^.

The increased popularity of medwakh smoking has prompted researchers around the globe to explore its association with several chronic conditions occurring in the oral cavity including cancers as well as those resulting in end-organ damage^6^. The polycyclic aromatic hydrocarbons (PAHs) released from medwakh smoke pose a higher risk than cigarettes and other tobacco products^6^. Previous studies have reported the acute effects of smoking medwakh on altering the systolic blood pressure and cardiovascular and respiratory systems^6,7^. Smoking medwakh results in the release of a mixture of toxic chemicals including nicotine, carbon monoxide, and oxidizing gases^7–9^. Inhalation of these toxic gases results in the triggering of oxidative stress pathways that leads to endothelial dysfunction, inflammation, and platelet activation^10,11^. As indicated above, within this context, saliva can be used as a diagnostic and monitoring tool for several illnesses with a high level of sensitivity and specificity^12^. Its benefits as a diagnostic tool include accessibility, the positive association between a number of indicators in serum and saliva, and the presence of both organic and inorganic components^13^. Numerous markers in saliva have been proposed to be used as diagnostic tests for several oral pathologies^14^ including cancer^15^. Salivary metabolite profiles have revolutionized the identification of several systemic and oral diseases like cancer and genetic abnormalities, viral (including SARS-CoV-2) and bacterial or fungal infections^16^. The development of various oral disorders, which are known to occur more frequently in smokers than non-smokers, is caused by the imbalance in the salivary redox mechanisms^17^.

Complementary to this, C-Reactive Protein (CRP) is one of the biomarkers that has strong potential for measurement in saliva for the diagnosis and/or monitoring numerous systemic conditions^18,19^ and oral illnesses^20^. Previous studies established a dose-response relationship between tobacco smoke exposure and salivary CRP levels^21^. Similarly, saliva is also a major rich source of enzymatic and non-enzymatic antioxidants that maintains the redox balance. Studies examining the level of antioxidants in smokers have reported an overall decline in the salivary total antioxidant capacity (TAC) in cigarette smokers compared to non-smokers^22^. According to reports, altering the glutathione metabolism is crucial for maintaining the redox equilibrium in cells, which is essential for the emergence and alleviation of ER stress. However, few studies have studied ALTP effect on oxidative stress and inflammation including medwakh.

In the present study, the salivary metabolomics profile of medwakh smokers is compared to non-smokers using HPLC-ESI-QTOF-MS in an effort to fill the knowledge gap regarding the physiological changes brought on by medhwakh smoking. The study also tries to establish a link with well known indicators of redox imbalance and eventually an inflammatory mechanism.

## Results

### Study design and participants’ characteristics

The study participants were UAE residents and a total of 40 subjects using different types of tobacco and nonsmokers were reruited in the pilot study. The study population comprised of 10 individuals from each group of smokers of only one type of tobacco such as medwakh, shisha or cigarettes and no other tobacco products. Non-smoking individuals were recruited as controls in the study. To explore the impact of medwakh smoking among young adults, we further recruited pure medwakh smokers (excluding the other types of smokers) and non-smokers. A total of 60 participants were finally recruited, divided into two equal groups, each having 30 non-smokers and 30 medwakh smokers (see supplementary table S1).

The medwakh smokers comprised of individuals who smoked more frequently than five times a day on average. The participants were all young adults of age between 19 and 25. The median age of 30 non-smokers was 23.5 (1), while the median age of 30 medwakh smokers was 24 (3). The results of the chi-square test and Maan-Whitney test performed to compare participants’ characteristics and age among the medwakh smokers and non-smokers respectively revealed a non-significant association except for smoking history (*p* < 0.001) and use of interdental aids (*p* < 0.05). We did not find significance (*p* = 0.851) when the median age of the medwakh smokers (24) was compared with non-smokers (23.5) (see supplementary Table S1. The experimental design, comprising the pilot and major studies, is provided, together with the methods employed and the parameters assessed (see supplemenetary Fig. S3).

### Metabolomic profile of the saliva in medwakh smokers

**Figure 1:**
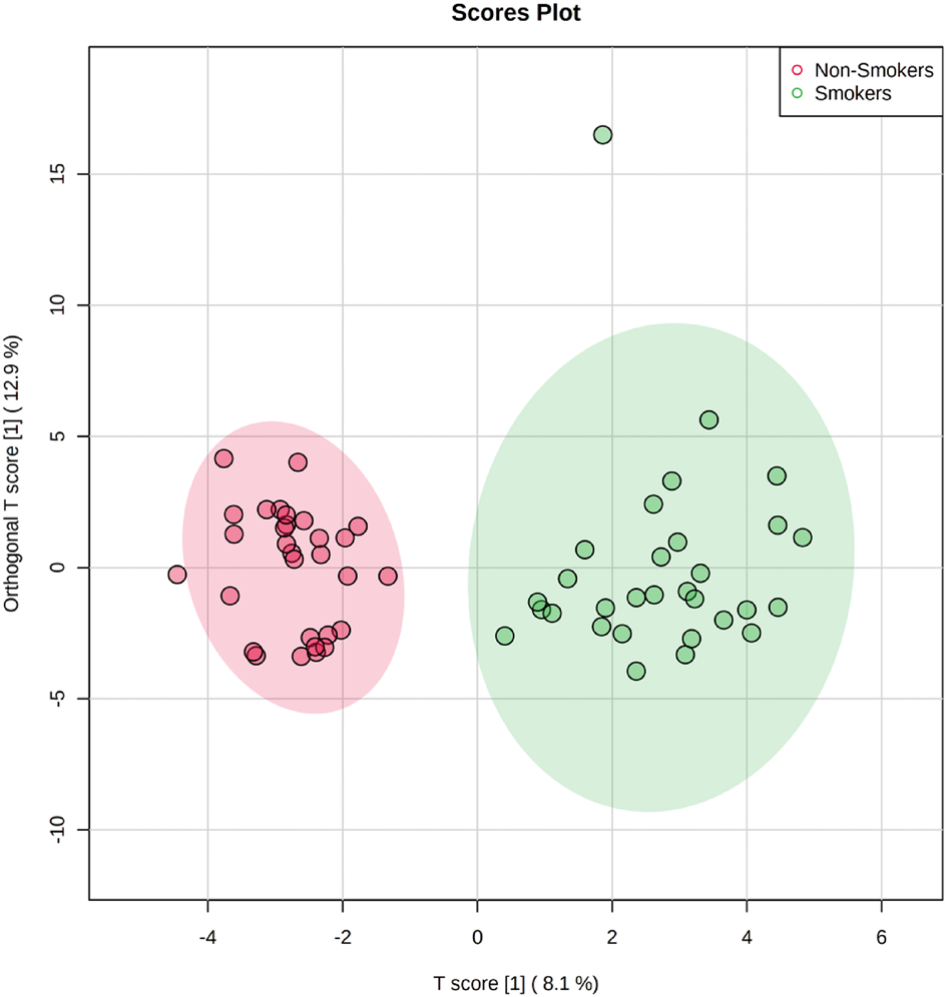
A well-defined alteration in the metabolomic profile of medwakh smokers was noticed using sparse partial least squares-discriminant analysis (sPLS-Da). The color red denotes non-smokers, while the color green denotes medwakh smokers.

Saliva samples from a total of 30 medwakh smokers and 30 non-smoking individuals were subjected to untargeted metabolomics analysis. Metabolites from the saliva samples of all the participants were analyzed and differential metabolite analyses were run independently among saliva samples of non-smokers and medwakh smokers. We identified a panel of 107 metabolites from the saliva samples of the study participants (2743/Data TrackID3313). All these differentially abundant metabolites were subjected to further downstream analysis and characterization to differentiate the medwakh smokers from the non-smokers. Data normalization was done by Shapiro Wilk test. Absolute amounts of 107 identified metabolites were further subjected to exploratory statistical evaluation. Among the 107 metabolites screened, 37 differed significantly in medwakh smokers in comparison to non-smokers with a statistically significant difference of *p* < 0.05 by parametric t-test. Clear differentiation between the study groups was observed by principal component analysis (PCA) (Fig. 1). The PCA scores plot for salivary metabolites revealed two distinct clusters representing the two study groups in the present study based on their smoking status.

### Differential characterization of medwakh smokers based on salivary metabolites

**Figure 2:**
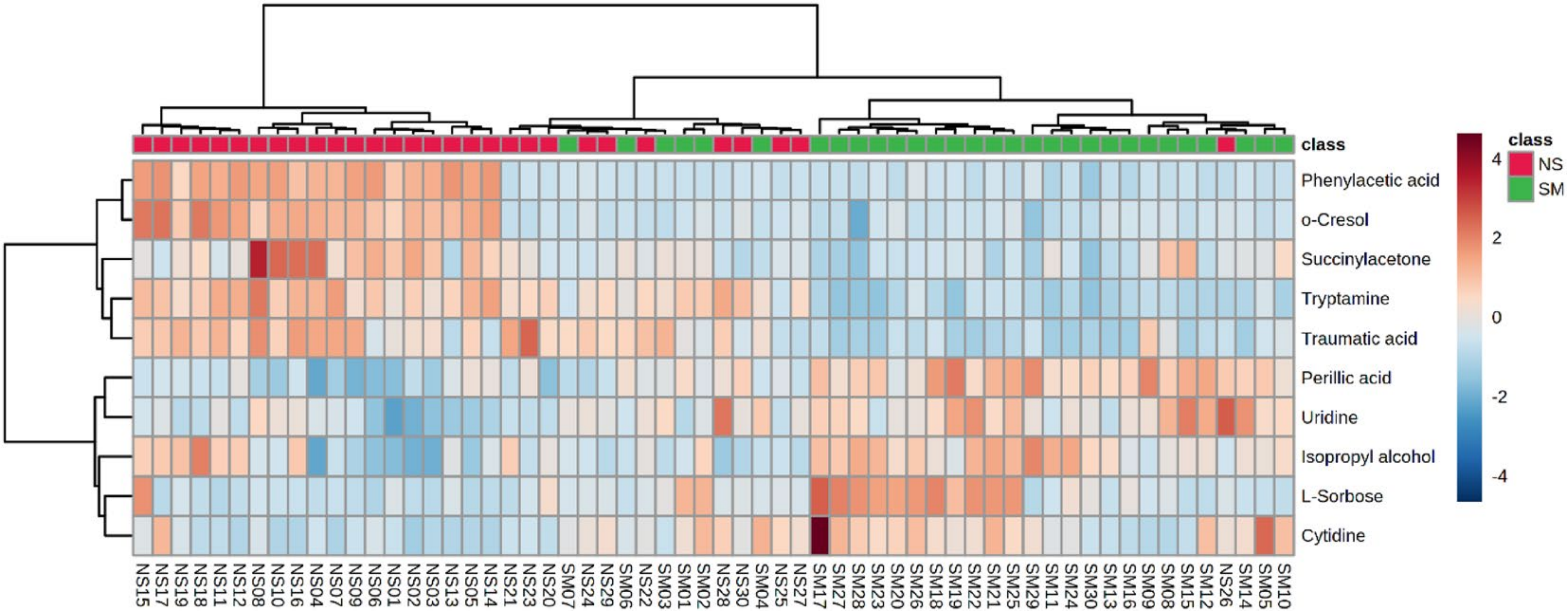
Heatmap with hierarchical clustering of metabolomics data with 107 absolutely quantified metabolites in the saliva samples of medwakh smokers and non-smokers. The heatmap was constructed with top 10 differential metabolites from the sparse partial least squared discriminant analysis (sPLS-Da).

Hierarchical clustering and heat map analysis of the most significantly abundant metabolites affected by medwakh smoking was performed. The 10 top-ranking metabolites from the partial least squared discriminant analysis are shown in Fig. 2. Heatmap visualization presented more noticeable metabolic changes owing to medwakh smoking.

The heatmap was constructed with the top 10 differential abundant metabolites from the sparse partial least squared discriminant analysis (sPLS-Da). NS-non-smokers and SM-smokers.

**Figure 3:**
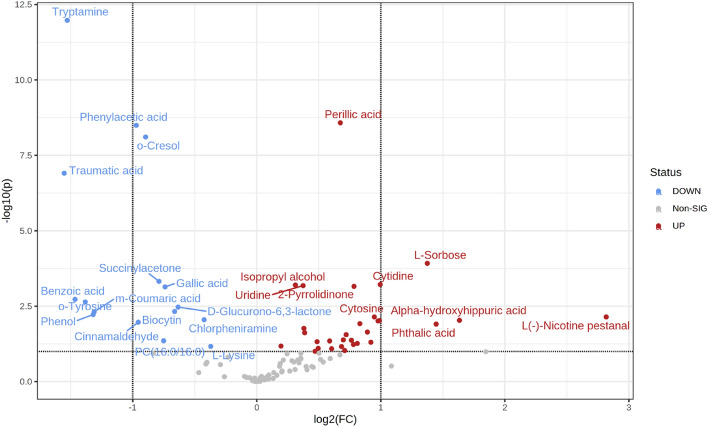
Volcano plot representing the metabolomics comparison between medwakh smokers and non-smokers. Most abundant and significantly altered metabolites in medwakh smokers are highlighted in red color whereas less abundant metabolites are shown in blue color.

The fold changes among the study groups were calculated using MetaboAnalyst 5.0 software. There were significant differences in absolute concentrations of 37 metabolites in medwakh smokers compared to non-smokers including many amino acids, nucleotides, organic acids, and carbohydrates or related substances (Table 1). A volcano Plot was further constructed to graphically represent the results of the t-test for the differential level of the unique metabolites (Fig. 3). The five most abundant metabolites of significance in the saliva of medwakh smokers identified by the Metaboanalyst software were L-sorbose, Cytidine, Alpha-hydroxyhippuric acid, Phthalic acid and L- Nicotine pestanal. Tryptamine, Phenyl acetic acid, o-cresol, Traumatic and Benzoic acids, on the other hand, was markedly less abundant in medwakh smokers.

**Table 1:** Significantly altered salivary metabolites in medwakh smokers in comparison to non-smokers. The fold changes among the study groups were calculated using MetaboAnalyst 5.0 software.

Metabolites	t.stat	*p*.value	Fold change	FDR
Less abundant metabolites in medwakh smokers				
m-Coumaric acid	2.9315	0.0048197	− 2.527	0.03034
o-Tyrosine	3.1908	0.0022909	− 2.436	0.01751
Tryptamine	9.0587	1.06E−12	− 2.406	1.1E−10
Phenol	2.853	0.0059935	− 2.403	0.03563
Benzoic acid	3.2588	0.0018736	− 2.25	0.01542
Phenylacetic acid	6.9776	3.2E−09	− 1.929	1.1E−07
Traumatic acid	6.0257	1.24E−07	− 1.9	2.7E−06
o-Cresol	6.7466	7.8E−09	− 1.774	2.1E−07
Biocytin	2.9382	0.0047304	− 1.654	0.03034
Gallic acid	3.5703	0.0007244	− 1.613	0.00646
Succinylacetone	3.7045	0.0004744	− 1.533	0.00646
D-Glucurono-6,3-lactone	3.056	0.0033876	− 1.404	0.02417
Chlorpheniramine	2.7078	0.0088847	− 1.255	0.04343
Most abundant metabolites in medwakh smokers				
Anserine	− 2.3182	0.004782	1.164	0.02328
L-Valine	− 3.6036	0.041431	1.184	0.11850
Isopropyl alcohol	− 3.6176	0.0006246	1.241	0.00646
Uridine	− 3.6016	0.0006568	1.295	0.00646
D-Alanine	− 2.0263	0.047341	1.377	0.14071
3-Hexenedioic acid	− 2.2581	0.027719	1.505	0.09567
L-Glutamic acid	− 2.0497	0.044921	1.528	0.13733
Perillic acid	− 7.0268	2.64E−09	1.568	1.1E−07
Pyroglutamic acid	− 2.5943	0.011983	1.606	0.04918
Niacinamide	− 2.0739	0.042539	1.608	0.13733
1,3-Dimethyluracil	− 2.0866	0.041332	1.622	0.13733
2-Pyrrolidinone	− 3.5836	0.0006948	1.693	0.00646
Cytosine	− 2.7875	0.007169	1.832	0.03837
L-Tryptophan	− 2.3402	0.022734	1.887	0.08388
Deoxyguanosine	− 2.0062	0.049501	1.924	0.14315
Cytidine	− 3.629	0.0006025	1.927	0.00646
Phthalic acid	− 2.5808	0.012409	1.954	0.04918
Cinnamic acid	− 2.6732	0.0097419	1.969	0.04343
Benzocaine	− 2.6816	0.0095271	1.984	0.04343
Alpha-hydroxyhippuric acid	− 2.6911	0.0092904	2.009	0.04343
L-Sorbose	− 4.1243	0.0001201	2.593	0.00214
L(-)-Nicotine pestanal	− 2.7873	0.0071725	7.0488	0.03837
Cinnamaldehyde	2.6362	0.01074	0.5155	0.04597
Uric acid	− 2.3182	0.023983	1.3067	0.08554

Most abundant and significantly altered metabolites in medwakh smokers are highlighted in red color whereas less abundant metabolites are shown in blue color.

### Untargeted metabolomics revealed alterations in metabolites level in medwakh smokers

**Figure 4:**
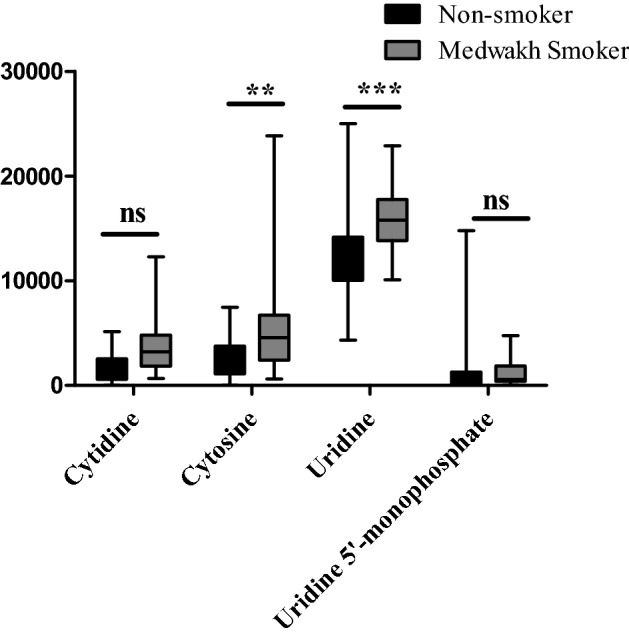
Differential salivary metabolites of top enriched pyrimidine pathway derived from the comparison between medwakh smokers and non-smoking controls. A higher fold-change was observed in the saliva of medwakh smokers for cytidine, cytosine, uridine and uridine 5’-monophosphate. Two-way analysis of variance (ANOVA) and post hoc Bonferroni multiple comparison analyses was applied to compare more than two independent variables. Samples included 30 medwakh and 30 non-smoking controls. ∗∗*p* < 0.01, ∗ ∗∗*p* < 0.001 is indicated. ns—not significant. Results are presented as median ± SEM.

When metabolomic enrichment analysis was done on the saliva samples of medwakh smokers, it demonstrated greater ratios of cytidine, cytosine, uridine, and uridine 5’- monophosphate when compared to non-smoking controls. The levels of cytosine (*p* < 0.01) and uridine (*p* < 0.001) were significantly enriched in medwakh smokers compared to non-smokers by two-way ANOVA analysis (Fig. 4). Similarly, we observed significant changes in the levels of certain essential metabolites in medwakh smokers such as L-sorbose (A), L-nicotine (B), alpha-hydroxy hippuric acid (C), traumatic acid (D), phthalic acid (E), D-alanine (F), L-glutamate (G) and uric acid (H); (see Supplementary Fig. S1).

A higher fold-change was observed in the saliva of medwakh smokers for cytidine, cytosine, uridine and uridine 5’-monophosphate. Two-way analysis of variance (ANOVA) and post hoc Bonferroni multiple comparison analyses was applied to compare more than two independent variables. Samples included 30 medwakh and 30 non-smoking controls. ∗∗*p* < 0.01, ∗∗∗*p* < 0.001 is indicated. ns—not significant. Results are presented as median ± SEM.

### Analysis of the metabolic pathways

**Figure 5:**
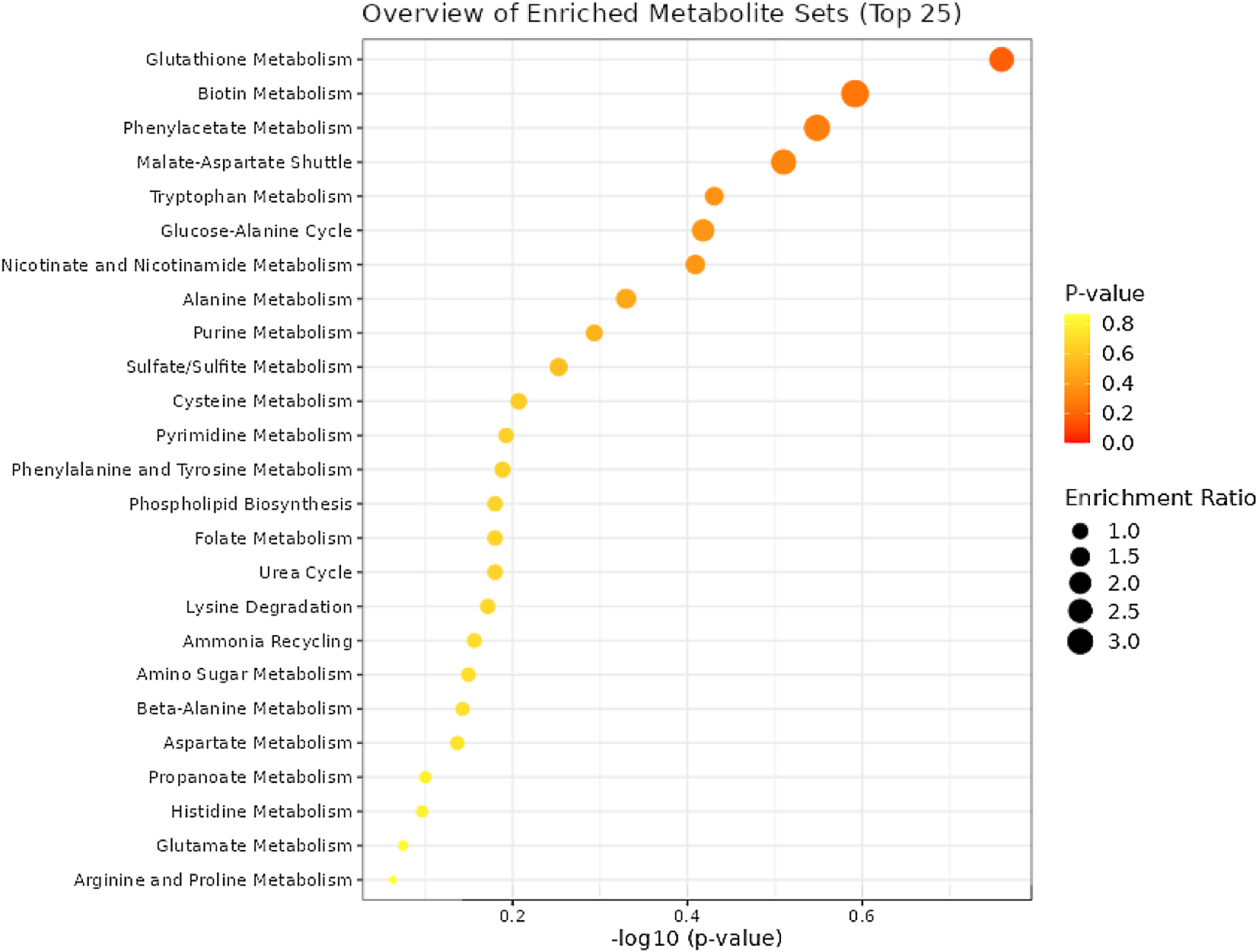
Metabolite Set Enrichment Analysis of top 37 differential metabolites derived from medwakh smokers in comparison to non-smoking controls. Top enriched pathways included glutathione metabolism, biotin metabolism, phenylacetate metabolism, malate aspartate shuttle, tryptophan metabolism, and nicotinate and nicotinamide metabolism pathways.

To further investigate the impact of medwakh smoking among the study population, metabolic pathways were analyzed using MetaboAnalyst 5.0 version. Significantly altered 20 metabolic pathways among the medwakh smokers were identified and the most relevant ones include linoleic acid metabolism (*p* = 0.099173) glutathione biosynthesis (*p* = 0.11195), D-glutamine and D-glutamate metabolism (*p* = 0.11783), ascorbate and aldarate metabolism (*p* = 0.15403), phenyl alanine (*p* = 0.18879), biotin (*p* = 0.18879), pyrimidine metabolism (*p* = 0.19129), tryptophan (*p* = 0.20651), nicotinate and nicotinamide metabolisms (*p* = 0.26974) (Fig. 5). A random sample from each group of the UHPLC-ESI-QTOF-MS base peaks chromatograms is shown in supplementary Fig. S2.

Top enriched pathways included glutathione metabolism, biotin metabolism, phenylacetate metabolism, malate aspartate shuttle, tryptophan metabolism, and nicotinate and nicotinamide metabolism pathways.

### Quantification of Glutathione peroxidase (GPx) and Total antioxidant capacity (TAC) of the saliva samples in medwakh smokers

**Figure 6:**
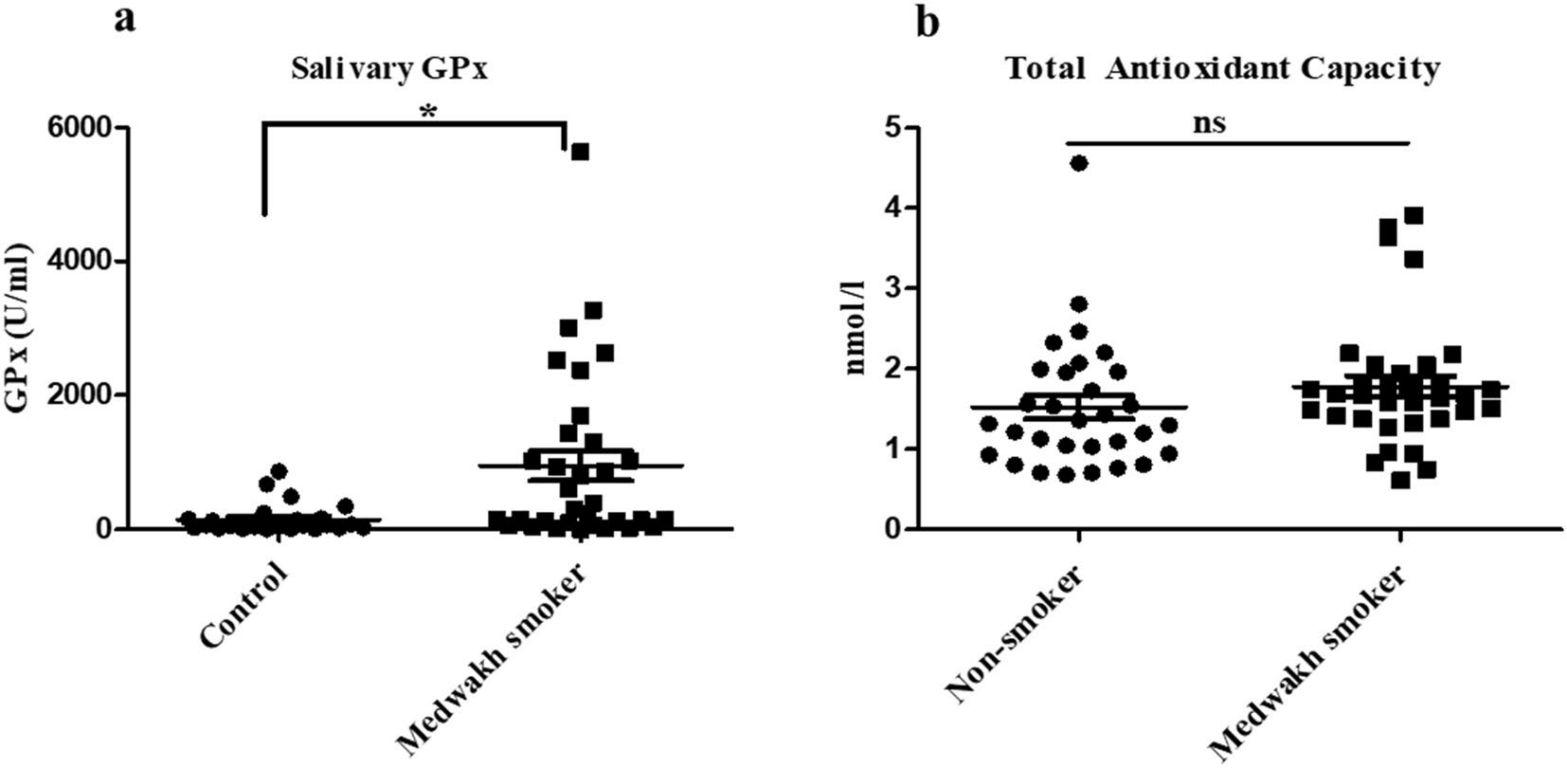
Salivary levels of (**a**) Glutathione peroxidase (GPx) and (**b**) Total Antioxidant Capacity (TAC) of saliva in medwakh smokers compared to non-smoking controls. Samples included 30 medwakh and 30 non-smoking controls. ∗*p* < 0.05 and ns-no significance by unpaired t-tests are indicated. Results are presented as mean ± SEM.

The results of the untargeted metabolomics analysis suggested that medwakh smoking may affect glutathione metabolism (Fig. 5). This led us to quantify the amounts of glutathione peroxidase (GPx), in the research samples in order to determine the extent of this alteration. Indeed, the salivary GPx levels among users of medwakh were found to be significantly higher compared to the non-smoking control group (995. 99 ± 99 vs 149.19 ± 208.70; *p* < 0.01; Fig. 6a). We initially speculate that greater salivary GPx levels in medwakh smokers could be explained by a compensatory rise to deal with the impending oxidant burden. However, the TAC levels did not differ significantly between medwakh smokers and non-smokers, and mean TAC levels for medwakh smokers were equivalent to non-smoking controls. (1.78 ± 0.76 vs 1.5 ± 0.79; Fig. 6b).

Samples included 30 medwakh and 30 non-smoking controls. ∗*p* < 0.05 and ns-no significance by unpaired t-tests are indicated. Results are presented as mean ± SEM.

### Salivary CRP levels

**Figure 7:**
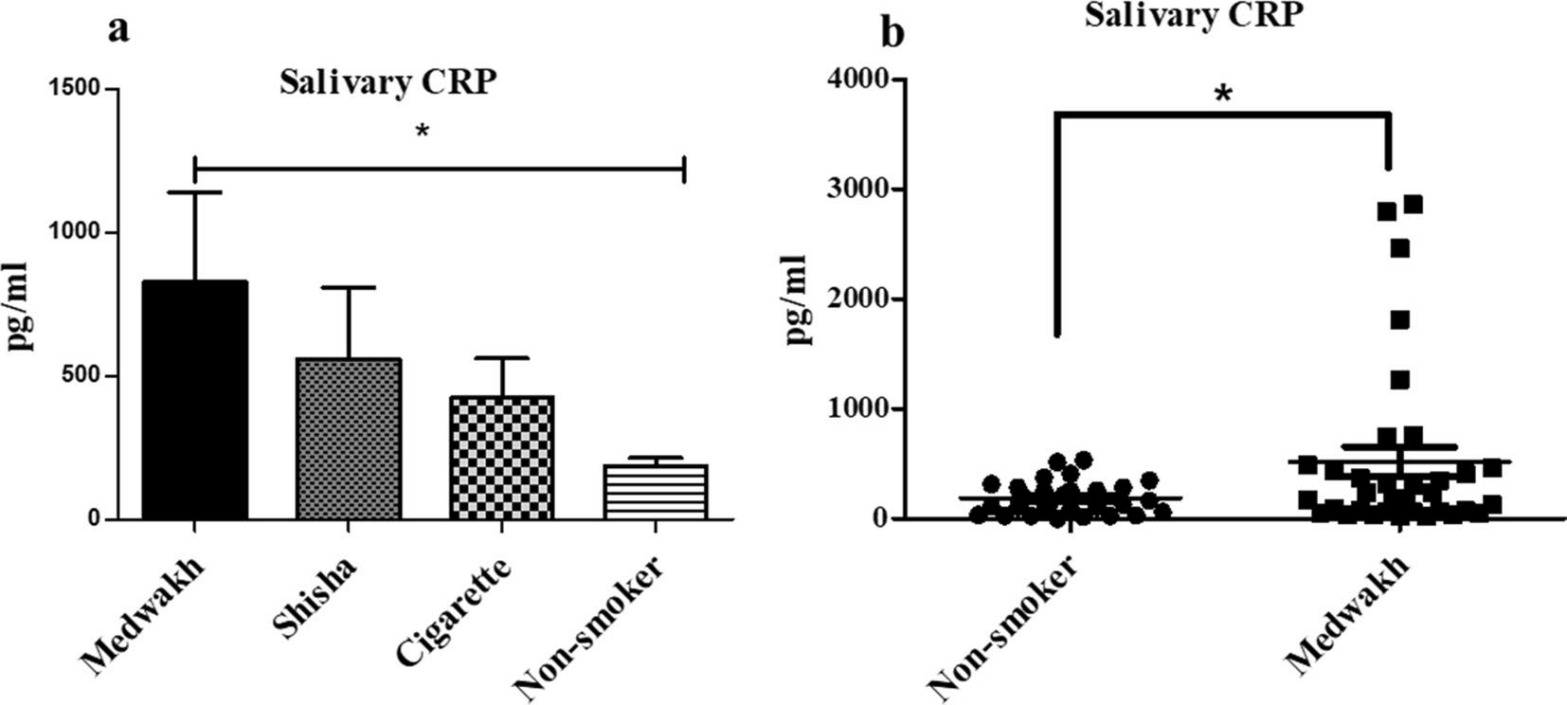
The salivary levels of C reactive protein (CRP) as an indicator of inflammation among users of different types of tobacco. (**a**) The levels of CRP among users of medwakh, shisha, and cigarettes in comparison with non-smoking controls in the pilot study. Saliva samples of medwakh smokers showed the highest level of CRP among other smokers with a significant difference compared to non-smokers (*p < 0.05). (**b**) Scatter plots showing the distribution of CRP levels in 30 medwakh smokers in comparison with 30 non-smokers. *p < 0.05 is indicated.

The findings here reported revealed among the medwakh smoker group an increased level of metabolites linked with inflammatory mechanism, such as pyroglutamic acid^23,24^, deoxyguanosine^25^ and anserine^26^. In order to investigate futher an eventual association of metwakh smoking and inflammation, we decided to measure CRP, a widely known clinical indicator of inflammatory conditions^27,28^. The levels of salivary CRP were first examined among 10 participants as a pilot study from each group of smokers and compared with non-smokers. The levels of salivary CRP were observed to be 826.928 ± 135.67, 558.56 ± 142, and 422.68 ± 110.14 pg/ml among users of medwakh, shisha, and cigarette respectively in the pilot study (Fig. 7a). Although the data was not significant, the levels of salivary CRP in medwakh smokers were the highest compared to shisha and cigarette smokers and significant with respect to non-smoking controls (*p* < 0.05) in this pilot study. Whereas when compared to non-smokers (N = 30), a significantly higher CRP was observed in medwakh smokers (N = 30); (519.3 ± 135.2 vs 186.7 ± 28.00 pg/mL; *p* < 0.05; Fig. 7b).

- The levels of CRP among users of medwakh, shisha, and cigarettes in comparison with non-smoking controls in the pilot study. Saliva samples of medwakh smokers showed the highest level of CRP among other smokers with a significant difference compared to non-smokers (*p < 0.05).
- Scatter plots showing the distribution of CRP levels in 30 medwakh smokers in comparison with 30 non-smokers. * p < 0.05 is indicated.

## Discussion

In the present study, a comprehensive investigation of metabolites extracted from the saliva samples of medwakh smokers along with non-smokers using UHPLC-ESI-QTOF-MS was performed. The untargeted salivary metabolite profiling revealed evidence of perturbation in nucleotide metabolites in medwakh smokers. Elevated levels of pyrimidine nucleotides and nucleosides in medwakh smokers point to changes in the purinergic signaling, which have been linked to the pathophysiology of salivary hypofunction^29^, a condition that often result in dry mouth or xerostomia.

The current study also indicated enrichment in biotin and tryptophan metabolism pathways in medwakh smokers. While Biotin is important for metabolic homeostasis^30^ maintaining gluconeogenesis, fatty acid, and amino acid metabolisms, tryptophan is largely implicated in the release of inflammatory signals associated with smoking and chronic obstructive pulmonary diseases (COPD)^31^. The significant decrease in biocytin observed in medwakh smokers is in coherence with a previous study in cigarette smokers and moist snuff users^32^. Tryptophan metabolism network is also associated with the catabolism of nicotinamide which is a precursor for the generation of the coenzymes NAD + and NADP + ^31^. The levels of nicotinamide were found to be significantly decreased in medwakh smokers. However, an increase in hydroxykynurenine, a toxic metabolite, capable of inducing oxidative damage and cell death is seen in medwakh smokers, although not significant. Smoking is the major exogenous source of oxidative stress species that disturbs the oxidative balance in the oral cavity^33^. Global metabolomic analysis of saliva, plasma, and urine samples revealed that exposure to cigarette smoke causes increased oxidative stress and inflammation as well as changes in redox homeostasis^34^. Tobacco smoke induced salivary oxidant generation causes morphological changes in the parenchyma of the salivary glands, decreased salivary secretion that contributes to biochemical alterations in the saliva^33^ increased inflammation^35^, changed protein carbonyls, and reduced enzyme activities^36^. The increased salivary CRP at levels reported in previous studies^28,37^ may serve as a plausible indicator of the inflammatory status of the medwakh smokers since we have excluded the effects of reported periodontal diseases among the study subjects as described in the exclusion criteria. Elevated levels of salivary CRP, a powerful biomarker of oral inflammation, may be a consequence of an oxidative burst caused by medwakh smoking. According to prior investigations^21^, elevated salivary CRP levels have been positively associated with both active and passive smoking in young, healthy individuals. Another interesting metabolite that exhibited a significant increase in medwakh smokers is Phthalic acid, an aromatic dicarboxylic acid. As an environmental chemical of heightened public concern, Phthalate causes a potential risk to male reproductive health^38^. The amount of Phthalate is significantly associated with reduced sperm concentration and suppressed androgen synthesis resulting in testicular dysgenesis raising a case of concern among medwakh smokers.

Cigarette smokers has increased GSH and glutathione peroxidase activities in their lung epithelial lining fluid compared with nonsmokers^39^ and these results are consistent with the high GSH levels observed in animals exposed to cigarette smoke^40^. Interestingly, in the present study, we observed an enrichment in glutathione metabolism in the saliva of medwakh smokers. This is supported by the significant presence of two important metabolites, L-glutamate and pyroglutamate (5-oxoproline) in the saliva samples of medwakh smokers. Increased levels of L-glutamate is reported to cause oxidative stress in the mammalian brain^41^ and 5-oxoproline is an easy measurable marker for oxidative stress resulting from cardiac injuries^42^. Glutathione (GSH) in conjunction with glutathione peroxidase (GSH-Px) and glutathione S transferase pi (GSTpi) maintain the redox balance and homeostasis in cells^43^. The enrichment in the glutathione metabolism pathway observed in the current metabolomics findings further correlates with our ELISA findings. We observed that the levels of salivary Gpx in medwakh smokers were significantly higher than in non-smokers. The increased anti-oxidant defense markers seen in medwakh smokers could be attributed to the young age of the cohorts recruited in the present study. To curb the huge amounts of reactive oxygen intermediates released from tobacco smoke, efflux in the anti-oxidant release as the body’s natural defense is expected. Likewise, the levels of anserine, another abundant antioxidant present in the saliva, are also found to be enhanced in medwakh smokers whereas gallic acid, which has both anti-inflammatory and anti-oxidant^44^ roles is found to be downregulated. However, when the total antioxidant capacity of the samples was analyzed, we did not find any difference. This is in contrast with previous reports in which a decline in the total antioxidant capacity among cigarette smokers is reported^22^. We presume that age has a profound influence on the abundance of metabolites in the present study, however, no gender differences could be cited as our samples were all from male medwakh users.

To the best of our knowledge, this study is the first to examine redox imbalance and inflammatory status in medwakh smokers. A well-established difference in the metabolism of medwakh smokers and alterations in potentially relevant pathways were identified. However, a more detailed analysis could be achieved by additional confirmation in the future by targeted analysis using synthetic standards. Furthermore, highly curated and annotated metabolic pathways could be developed to identify specific tobacco-derived carcinogens In addition, owing to the difficulty in identifying pure medwakh smokers as majority of the smoking population prefer mixed tobacco products, the present metabolomics analysis was performed on a relatively small number of subjects (N = 60) resulting in reduced ability to perform any stratification. Similarly, use of only male subjects pose another limitation to the study, although gender-specific cohort could avoid confounding factors that might complicate the metabolomics data analysis. Likewise, the possibility of BMI in determining the anti-oxidant and inflammatory capacities of medwakh smokers was not addressed in the current study. However, given the participants’ youth, this kind of supplementary data might not be as pertinent as it would be for an older cohort (age 45–50 year old). Additionally, it could be argued that under these circumstances changes in saliva concentrations do not preserve ratios in a linear fashion when it becomes more diluted in non-smokers or more concentrated specifically in case of dry mouth among smokers, as was mentioned above in this study where the metabolites were extracted from 100 µL of participant saliva. We have decided not to attempt any volume normalization or reference metabolite normalization, as advised in other body fluids, despite the fact that the natural fluctuation concentrations may be disputed as a confounding factor. The majority of the metabolites (70) between smokers and non-smokers remained unaltered, which supports the idea that the changes presented here reflect the mouth's chemical environment at the time the samples were taken. Nevertheless, significant differences in the levels of the metabolites detected in the small cohort and the consistency of findings on salivary CRP and anti-oxidant parameters with previous studies support the robustness of the method and the need to use this approach on a larger study. Taken together, the present study provides a comprehensive understanding of the impact of medwakh smoking on increasing levels of metabolites like nicotine, GSH and increased glutathione peroxidase activities. Metabolites like L-glutamate, 5-oxoproline, glycine, and alanine, which are linked to ER stress; guanosine, cytidine, and urate, which are by-products of nucleotide degradation; and hippurate, which is produced during the metabolism of proteins and amino acids; and hydroxykynurenine suggest the modulation of oxidative stress and redox balance in medwak smokers.

The suggestion that using these cigarette substitutes is a better option is alarming since this practice frequently deceives the public, especially young people. The authors consider it a wake-up call that urgently requests a global awareness focusing on the detrimental aspects of medwakh smoking especially markers associated with redox homeostasis and CRP and its possible deleterious effect on male reproductive system.

## Methods

### Population and study design

The data on study variables were collected at a single point of time using a cross-sectional quantitative study methodology. The design and nature of the study were explained to the participants and informed written consent for their participation was procured. In addition, the participants were requested to complete a questionnaire that included demographic information such as overall health, periodontal condition, medwakh use, and smoking history (see supplementary Table S1). The study procedure was approved by the University of Sharjah's Research Ethics Committee (REC-18-10-23-01) in accordance with national and international norms, including the Helsinki Declaration. All methods and procedures were performed in accordance with the relevant guidelines and regulations. A chi-square test and Maan-Whitney test were conducted to compare participants’ characteristics and age among the medwakh smokers and non-smokers respectively.

The inclusion criteria were the following; young individuals who smoked just one type of tobacco product such as medwakh, shisha, or cigarettes and no other tobacco products, and those who had never smoked. We excluded subjects with signs of periodontitis such as clinical attachment loss and sulcus depth > 3mms and those who under periodontal treatments, antibiotics, or steroid therapies during or within 3 months of the trial, as well as those who were undergoing orthodontic treatment. Medically compromised participants and those with chronic inflammatory diseases and pregnant female participants were also excluded. Since the vast majority of smokers encountered for the use of each tobacco product were males, the participants enrolled were all males in the current study.

### Saliva collection

Participants were instructed to observe a two-hour fast without eating, smoking or drinking followed by a thorough rinsing of the mouth to prevent any sample contamination. Passive drooling was used to gather participants' saliva. Participants were seated with their head tilted forward, allowing saliva to pool in front of the mouth. The samples were collected in sterile collection containers and immediately transported to the laboratory in ice storage boxes. To reduce multiple freeze–thaw cycles, remove cell debris and mucus, samples were initially centrifuged at 2500 rpm for 5 min, aliquoted and stored at – 80 C freezer. Saliva samples were stored in -80C until the required number of samples (N = 60) was achieved. On the day of the metabolomics analysis, saliva samples were thawed in ice and further centrifuged at 10,000*g* for 10 min at 4 C. The supernatants were collected and used in the study.

### Metabolites extraction and sample preparation

Saliva samples were thawed at room temperature and 100 μL of the samples from each group were mixed with 300 μL methanol (≥ 99.9%, LC–MS CHROMASOLV) for protein precipitation, vortexed and incubated at − 20 ◦C for 2 h. The samples were vortexed again, centrifuged at 14,000 rpm for 15 min and supernatants were transferred to new glass vials. The supernatants were evaporated in a SpeedVac, EZ-2 Plus (GeneVac, Ipswich, UK) at 35–40 °C. To prepare the extracts for LC-MS/MS analysis, they were first resuspended in 200 µl deionized water containing 0.1 percent formic acid (LC-MS CHROMASOLV, Honeywell, Seelze, Germany) and vortexed for 2 min for complete mixing. The extracts were then filtered using a 0.45 µm hydrophilic nylon syringe filter for LC-MS/MS analysis. Equal volumes (10 µl) of 30 samples from each study group were collected and pooled for the quality control (QC) and placed in the autosampler at 4℃ to analyze the reproducibility of the analysis.

### Ultra-high-performance liquid chromatography coupled to electrospray ionization and quadrupole time-of-flight mass spectrometry (UHPLC-ESI-QTOF-MS)

An ultra-high-performance liquid chromatography system (UHPLC) (Bruker Daltonik GmbH, Bremen, Germany) was used in conjunction with a quadrupole time-of-flight mass spectrometer for the LC-MS/MS analysis (QTOF). The system was equipped with an electrospray ionization (ESI) source, a solvent delivery systems pump (Elute UHPLC HPG 1300), an autosampler, and a thermostat column compartment. Windows 10 Enterprise 2016 LTSB was used as the computer operating system. The computer operating system was Windows 10 Enterprise 2016 LTSB, and the software used was Bruker Compass HyStar 5.0 SR1 Patch1 (5.0.37.1), Compass 4.1, Version 6.2. Two different mobile phases were utilized: one with water and 0.1 percent formic acid (A) and the other with acetonitrile and 0.1 percent formic acid (B). The gradient program followed was: 0–2 min, 99% A: 1% B; 2–17 min, 99–1% A: 1–99% B; 17–20 min, 99% B: 1% A and the flow rate was fixed at 0.25 mL/min. Subsequently, 20–20.1 min 99% B to 99% A; 20.1–28.5 min, 99% A: 1% B at 0.35 mL/min flow rate; 28.5–30 min; 99% A: 1% B at 0.25 mL/min. Hamilton^®^ Intensity Solo 2 C18 column (100 mm × 2.1 mm × 1.8 m) was employed for the separation. The oven temperature was maintained at 35 °C.

The drying gas flow rate was 10.0 L/min (220 °C), the capillary voltage was 4500 V, and the nebulizer pressure was 2.2 bar in the ESI. The collision energy ranged from 100 to 250% and was set at 20 eV with a 500 V End Plate offset for the MS2 acquisition. For the external calibration, sodium formate was used. For the calibrant sodium formate, the auto MS scan segment used for acquisition ranged from 0 to 0.3 min, and for auto MS/MS, it ranged from 0.3 to 30 min.The acquisition was done in positive mode at 12 Hz in both segments, and the automatic in-run mass scan range was 20 to 1300 m/z. With a cycle length of 0.5 s and a threshold of 400 cts, the width of the precursor ion was 0.5 and the number of precursors was 3. After three spectra, the active exclusion was removed and released after 0.2 min.

The collision energy stepping for MS2 was set at 20 eV and varied between 100 and 250%. Mass calibration was done prior to analysis according to the manufacturer’s recommendations using external mass calibration (10 mM sodium formate calibrant solution). The analysis was performed using a randomized sequence order with five injections of solvent A (0.1% formic acid in deionized Water) sample at the beginning of the sequence for apparatus equilibration, followed by five injections of the pooled QC sample. Additionally, one QC injection was performed every (9–10 samples) to minimize the carryover and evaluate the reproducibility of the analysis and monitor batch effects as described by Wehrens et al.^45^. A full description of data processing including filtering thresholds, MV thresholds and QC metrics by following a reported template on QC reporting for metabolomics^46^ is given the supplementary information (see supplementary methods).

### Data processing and analysis

The MS data (Bruker Daltonics, Billerica,MA, USA) was preprocessed through the the MetaboScape^®^ 4.0 program.The T-ReX 2D/3D approach employed the peak area to quantify the feature after bucketing the processed data with an intensity threshold of 1000 and a peak length of 7 spectra. The calibration for mass spectra was done in 0–0.3 min and chromatographic peak widths less than 0.03 min were not included in the analysis. The auto MS/MS scan was performed using the average method, with scan parameters ranging from 0.3 to 25 min of retention time and 50–1000 m/z of mass. LC-MSMS-QTOF was used to examine all of the study samples in duplicates, yielding a data set of 3887 characteristics across the 60 samples of the two groups used in the study.Unreliable features were removed using the QC samples. The HMBD 4.0 software was used to map the MS/MS spectra and retention time to identify the metabolites.The selected metabolites were filtered by picking a higher annotation quality score that indicates the MS/MS score, m/z values, best retention time, mSigma, and analyte list spectrum library, and the annotation procedure was followed to identify the compounds with MS/MS using library matching.The peak intensity of each metabolite obtained was used for quantification of the data matrix and only significant compounds with a *p*-value of < 0.05 were selected from HMDB 4.0. Following filtration, a total of 107 different metabolites were chosen. After that, the data was saved as a CSV file and imported into MetaboAnalyst 5.0. (Mcgill University, Montreal, QC, Canada). Most significant discriminating features were selected from the study groups by performing sPLS-DA using MetaboAnalyst and the samples were classified accordingly. Multiple hypothesis testing was done, and the number of false positives was reduced, using the false discovery rate (FDR) method.

### Measurement of GPx activity and total antioxidative capacity in saliva

The GPx activity in the saliva samples was determined using the GPx assay kit (Human Glutathione Peroxidase 1 sandwich ELISA Kit (GPX1), Cat no# RK09254, Abclonal, USA) according to the manufacturer’s instructions. 1 U (μmol/min) of GPx activity is considered as the amount of enzyme that catalyzes the conversion of one micromole of substrate per minute. Total Antioxidant Capacity Assay Kit (Cat no# ab65329, Abcam, USA) was used to quantify TAC in saliva samples according to the manufacturer's protocol to assess antioxidant capacity utilizing an oxidation–reduction colorimetric assay at 570 nm wavelength. TAC level provides an overall total antioxidant capacity in the samples which may give more relevant biological information compared to that obtained by the measurement of individual antioxidant components present in the samples.

### Quantification of salivary C reactive protein (CRP)

The levels of CRP in the study samples were quantified by an indirect sandwich ELISA using a salivary C reactive protein assay kit (Salimetrics, USA) following the manufacturer’s instructions. Briefly, 150 µl of 1:2 diluted saliva samples were added into anti-CRP capture antibody pre-coated plates which were bound by the anti-CRP detection antibody linked to horseradish peroxidase. The CRP levels were measured by the reaction of the horseradish peroxidase (HRP) enzyme to the substrate tetramethylbenzidine (TMB) which was detected on a standard plate reader at 450 nm.

### Metabolic pathway analysis and statistical methods

Metabolic pathways and enrichment analysis (MSEA) were processed using MetaboAnalyst 5.0. The levels of observed metabolites were normalized using MetaboAnalyst software. Unpaired t-tests were performed to study the statistical significance among the study groups (non-smoker vs medwakh smoker) using MetaboAnalyst 5.0 and Prism (v5; GraphPad Software). To compare participants’ characteristics among the medwakh smokers and non-smokers, a chi-square test was conducted, and Mann–Whitney U test was performed to examine the difference in age among the medwakh smokers and non-smoking controls. Two-way ANOVA with Bonferroni post hoc tests and unpaired t-tests were conducted using graph pad prism software to examine the significance of the abundance of various metabolites. A *P*‐value of < 0.05 was considered statistically significant.

## Supplementary Information

Supplementary Information 1.

Supplementary Information 2.

## Data Availability

Data is contained within the article or Supplementary Material.
