# Supplementary material for: Effects of medwakh smoking on salivary metabolomics and its association with altered oral redox homeostasis among youth
Source: Sci Rep. 2023 Feb 1;13:1870. doi: 10.1038/s41598-023-27958-w (PMC9891755; doi:10.1038/s41598-023-27958-w)
Supplement: Supplementary file 1 — Supplementary Information 1. [file 41598_2023_27958_MOESM1_ESM.docx]

Pooling the same volume (10uL) of each sample enabled the preparation of the quality control (QC) sample (control and test). In order to track the experiment's stability and batch effects, the QC sample was injected at regular intervals (every 9–10 samples), and both the control and test samples were evaluated by LC–QTOF in duplicate.

The performance of the column reversed-phase liquid chromatography (RPLC) separation, multipoint retention time calibration, and the mass spectrometer were tested using a test mixture of TRX-2101/RT-28-calibrants for Bruker T-ReX LC-QTOF ( Nova Medical Testing Inc.). TRX-3112-R/MS Certified Human serum for Bruker T-ReX LC-QTOF solution from Nova Medical Testing Inc. was prepared from pooled human blood to check the performance of the LC-MS instruments. As a standard protocol, the test mixture data files were uploaded to Metaboscape 4.0 and confirmed the presence of the entire set of metabolites in the samples.

The m/z measurements were externally calibrated using 10 mM of sodium formate before sample analysis. In addition, sodium formate solution was injected at the beginning of each sample run and used for internal calibration during data processing. Raw data was analysed with the software MetaboScape 4.0 (Brüker Daltonics, Germany) using the TReX (Time aligned Region complete eXtraction) algorithm. All samples’ peak lists were aligned in a single bucket table. The peak intensities of m/z values must be more than 1000 in at least 30 injections in order to be considered in the bucket table. If not, the m/z values were automatically removed from the bucket table as they were most likely to be experimental artifact of some sort, and thus non-informative.

Bucketing parameters for peak detection of the processed data in T-ReX 2D/3D workflow were as follows: intensity threshold equal to 1000 counts along with minimum peak length of 7 spectra. The parameters for data bucketing were assigned with a retention time range starting at 0.3 min and ending at 25 min for each single run, while the mass range started at 50 m/z and ended at 1300 m/z, and the MS/MS import method was done using the average spectrum out of all MS/MS spectra. After the formation of the bucket table, the annotation of metabolites was done based on mapping the MS/MS spectra and retention time in the HMBD 4.0. All features having MS/MS values were further selected to be annotated by HMDB 4.0.

The QC samples were considered as a benchmark for further selection and sorting of the identified metabolites in the study samples. Metabolites that displayed a relative standard deviation (RSD) more than 30% in comparison to QC samples were rejected. The metabolites having mass error more than 5 ppm were also rejected. Then, the selected metabolites were filtered by choosing the set with a higher annotation quality score (AQ score) representing the best retention time values, MS/MS score, m/z values, mSigma.

The average intensities of the identified metabolites were further taken from 2 injections of each sample and for samples having zero value (missing value) in any injection, we have taken the maximum peak value.

For the statistical analysis of the data, the filtered metabolites were uploaded to the Metaboanalyst software. Data normalization was then performed followed by parametric t-test analysis to statistically analyze the data.
