# Supplementary material for: Effects of medwakh smoking on salivary metabolomics and its association with altered oral redox homeostasis among youth
Source: Sci Rep. 2023 Feb 1;13:1870. doi: 10.1038/s41598-023-27958-w (PMC9891755; doi:10.1038/s41598-023-27958-w)
Supplement: Supplementary file 2 — Supplementary Information 2. [file 41598_2023_27958_MOESM2_ESM.docx]

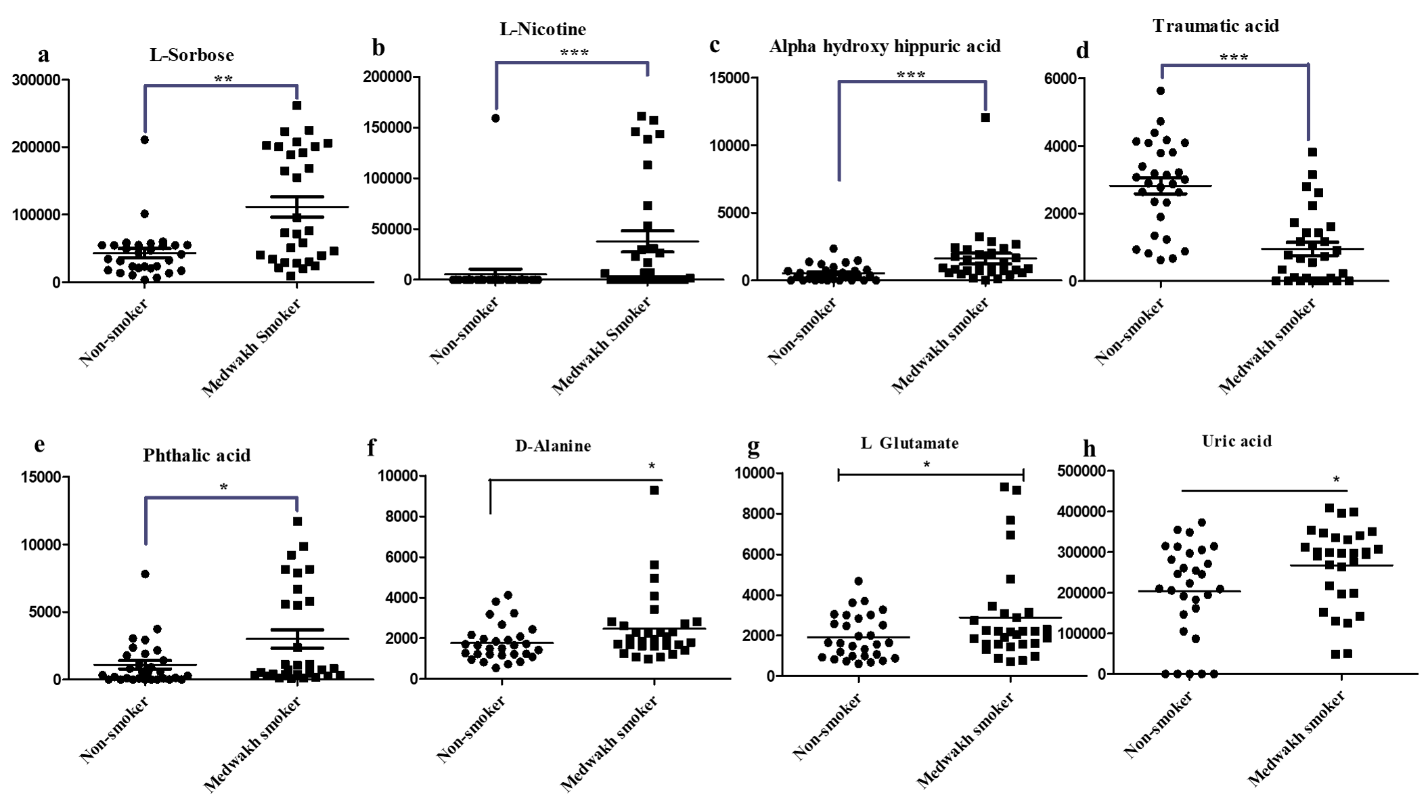


**Supplemetary Figure S1: Differential salivary metabolites of top enriched pathways derived from the comparison between medwakh smokers and non-smoking controls.** A higher fold-change in metabolites profile was observed in the saliva of medwakh smokers. Unpaired t-tests were applied to compare the two independent variables. Samples included 30 medwakh and 30 non-smoking controls. ∗p < 0.05; ∗∗p < 0.01, ∗∗∗p < 0.001 are indicated. Results are presented as median ± SEM.


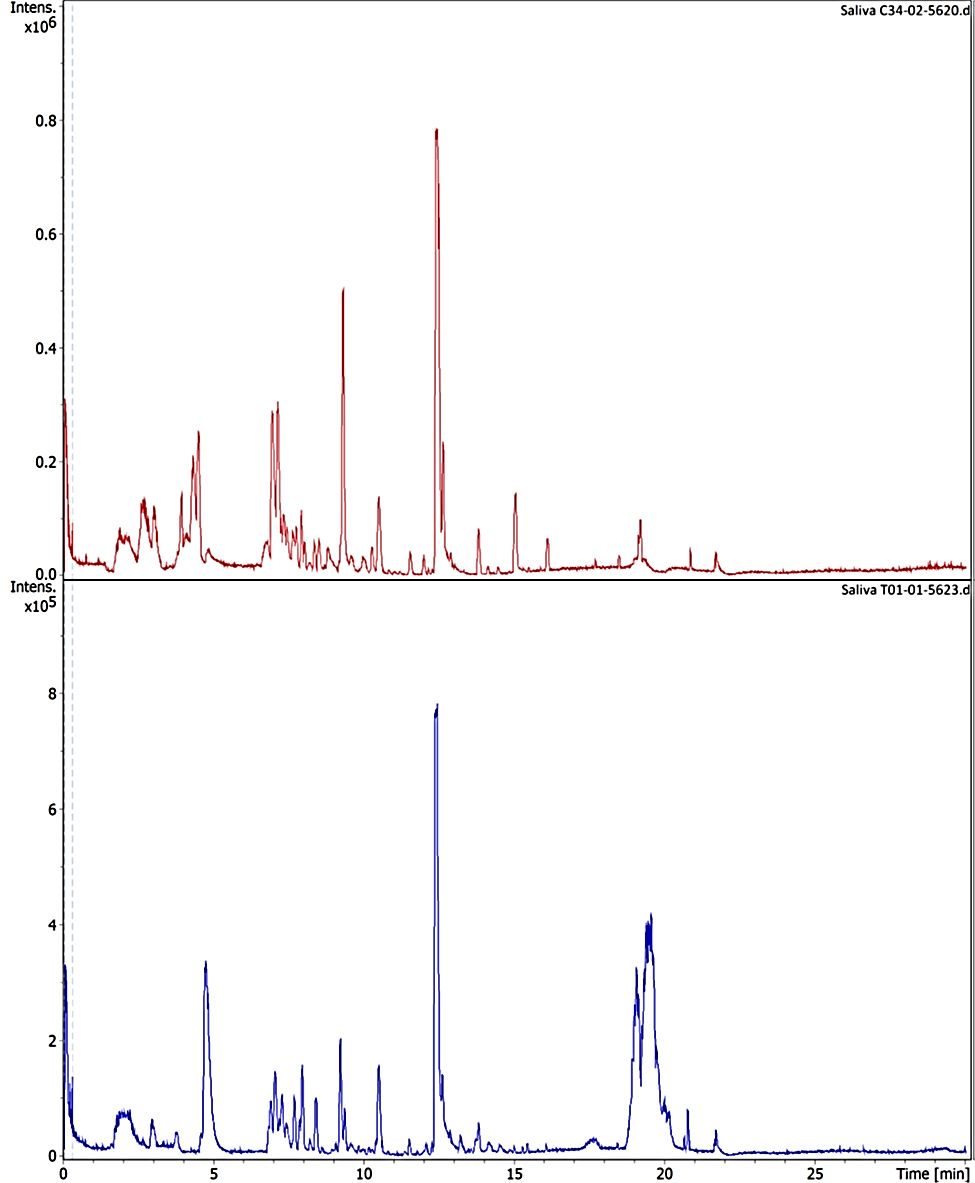


**a**

**b**

**Supplementary Figure S2: Positive ion base peak intensity chromatograms obtained from the analysis of (a) non-smoker and (b) medwakh smoker saliva samples.**

**
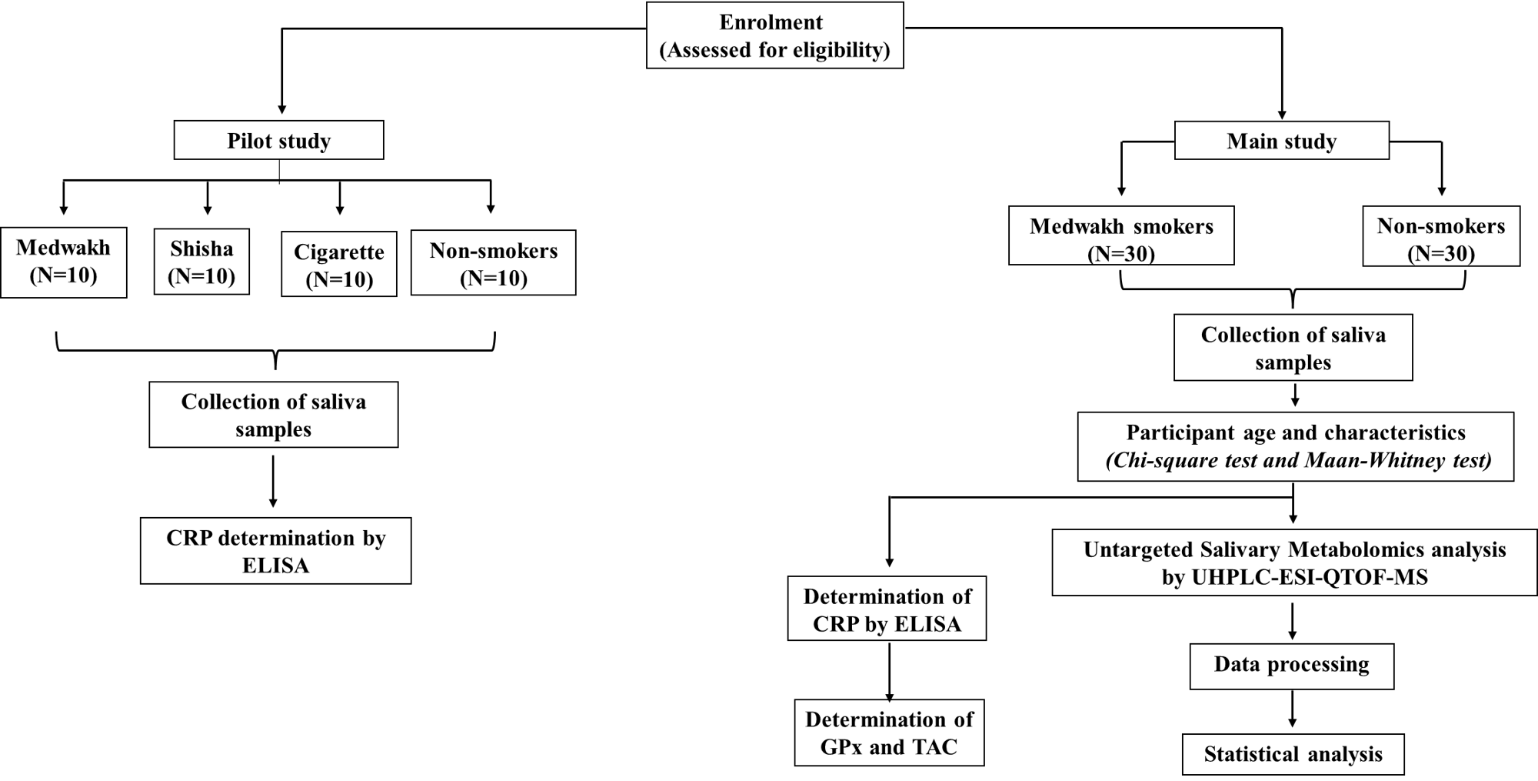
**

**Supplementary Figure S3: Experiment scheme detailing the methodology used and parameters evaluated in the untargeted metabolomic profiling of medwakh smoker saliva samples.**

**Supplementary Table S1: Demographics of the study participants divided into two groups based on smoking status.** Chi-square test and Mann-Whitney test were conducted to compare participants’ characteristics and age among the medwakh smokers and non-smokers respectively. p<0.05 is considered as statistically significant.

|  |  |  |  |  |  |  |
| --- | --- | --- | --- | --- | --- | --- |
| **Characteristics** | **Categories** | **Medwakh** | | **Non-smoker** | | ***p*-value** |
|  |  | n | % | n | % |  |
| Age | Median, IQR | 24 ( 3) | | 23.5 ( 1) | | .851 |
|  |  |  |  |  |  |  |
| Education level | Intermediate | 1 | 3.3% | 0 | 0.0% | .313 |
|  |  |  |  |  |  |  |
|  | High (university degree) | 29 | 96.7% | 30 | 100.0% |  |
|  |  |  |  |  |  |  |
|  |  |  |  |  |  |  |
| Smoking history | Yes | 29 | 96.7% | 0 | 0.0% | <.001 |
|  |  |  |  |  |  |  |
|  | No | 1 | 3.3% | 30 | 100.0% |  |
|  |  |  |  |  |  |  |
|  |  |  |  |  |  |  |
| Tooth brushing frequency | at least twice daily | 19 | 63.3% | 20 | 66.7% | .962 |
|  |  |  |  |  |  |  |
|  | once a day | 10 | 33.3% | 9 | 30.0% |  |
|  |  |  |  |  |  |  |
|  | less frequently | 1 | 3.3% | 1 | 3.3% |  |
|  |  |  |  |  |  |  |
| Interdental aid use | daily | 6 | 20.0% | 15 | 50.0% | .037 |
|  |  |  |  |  |  |  |
|  | sometimes | 8 | 26.7% | 7 | 23.3% |  |
|  |  |  |  |  |  |  |
|  | never | 16 | 53.3% | 8 | 26.7% |  |
|  |  |  |  |  |  |  |
